# Edible fungi crops through mycoforestry, potential for carbon negative food production and mitigation of food and forestry conflicts

**DOI:** 10.1073/pnas.2220079120

**Published:** 2023-03-13

**Authors:** Paul W. Thomas, Alistair S. Jump

**Affiliations:** ^a^Faculty of Natural Sciences, University of Stirling, Stirling FK9 4LA, UK; ^b^Mycorrhizal Systems Ltd., Lancashire PR25 2SD, UK

**Keywords:** ectomycorrhizal, sustainability, forestry, land-use conflict, mycoforestry

## Abstract

Demand for agricultural land is a potent accelerating driver of global deforestation, presenting multiple interacting issues at different spatiotemporal scales. Here we show that inoculating the root system of tree planting stock with edible ectomycorrhizal fungi (EMF) can reduce the food-forestry land-use conflict, enabling appropriately managed forestry plantations to contribute to protein and calorie production and potentially increasing carbon sequestration. Although, when compared to other food groups, we show that EMF cultivation is inefficient in terms of land use with a needed area of ~668 m^2^ y kg^−1^ protein, the additional benefits are vast. Depending on the habitat type and tree age, greenhouse gas emissions may range from −858 to 526 kg CO_2_-eq kg^−1^ protein and the sequestration potential stands in stark contrast to nine other major food groups. Further, we calculate the missed food production opportunity of not incorporating EMF cultivation into current forestry activities, an approach that could enhance food security for millions of people. Given the additional biodiversity, conservational and rural socioeconomic potential, we call for action and development to realize the sustainable benefits of EMF cultivation.

Many of the world’s forests are highly degraded, and despite significant afforestation activities, net loss of forest area remains high at some 4.7 million hectares per year (data for 2010 to 2020) (1). Demand for agricultural land is the biggest driver of global deforestation, and this is forecast to accelerate (1).

Deforestation presents multiple interacting issues at different spatiotemporal scales. Forests house much of terrestrial biodiversity (1), sequester ~2 Gt carbon per year, and ~75% of the worlds accessible fresh water arises from their watersheds (2). At the same time, climate change presents an accelerating risk to food production systems. Increased extreme weather events, trends toward less predictable weather cycles that reduce harvest yields, and climate change-driven desertification all negatively impact agricultural output, risking the need for more land to even maintain existing levels of agricultural production.

Pathways to reduce this inherent land-use conflicts exist. Agroforestry, involving the addition of trees to agricultural landscapes or farming in forest landscapes, is one well-established approach (3), but such systems do not recreate natural forests and economics typically favor monocultures with commercial agricultural practices. However, the cultivation of edible ectomycorrhizal fungi (EMF) (4–6) in mycoforestry presents a promising emerging technology that combines food production with afforestation while meeting conservation and biodiversity priorities.

EMF are plant symbionts, exchanging nutrition for plant-derived carbohydrates through a structure with the host plants root system known as mycorrhiza_,_ and include over 900 edible species (6). Since the 1970s, the Périgord truffle (*Tuber melanosporum*), a climate sensitive Mediterranean EMF, has been widely and successfully cultivated (7, 8). Aside from the *Tuber* genus, cultivation of EMF has been an under-researched field, and although progress has been made with the cosmopolitan *Suillus* and *Astraeus* genera (5), the greatest advance has been in the widely distributed *Lactarius* genus (Fig. 1) and mostly with the appreciated European *Lactarius deliciosus*. Experimentation with *L. deliciosus* presents annual yields of up to 1,089 kg ha^−1^, dependent on the methods employed (5). Additional experimentation has also been successful in France (9) and in New Zealand, where a markedly higher production figure of 3,000 kg ha^−1^ has been reported, although this latter report is lacking in detail (10). Nevertheless, the figure of 1,089 kg ha^−1^ gives a good indication as to what is possible (5), and as the field is expanded, it is expected that more species will be identified with different bioclimatic preferences and yield potentials. Although the field is nascent, it shows great promise and a full roadmap has recently been published suggesting implementation in the neotropics, to combine biodiversity and conservational goals with food production and afforestation using *Lactarius indigo* (4). The harvesting of EMF can play an important role in rural socioeconomic development by providing a significant food source (4, 6). Another potential major benefit is carbon sequestration and the ability to help mitigate anthropogenically driven greenhouse gas (GHG) emissions and the resulting impact on the climate (4–6). Proposed EMF cultivation involves tree planting as orchard-like systems (5) or methods closer to afforestation with conservational goals (4), but in either method understanding of the GHG flux of the plant-partner component was previously unknown.

**Fig. 1.**
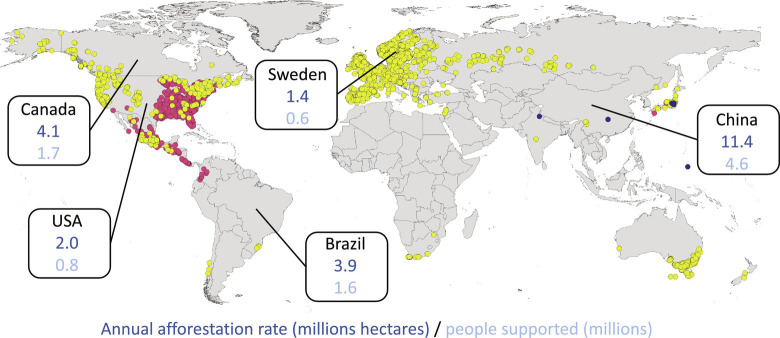
Global distribution of three *Lactarius* species with cultivation potential. *L. deliciosus* (yellow)*, L. indigo* (pink)*, and L. subindigo* (blue) compiled from georeferenced records from the Global Biodiversity Information Facility (11). Annual afforestation activity for the period 2010 to 2020 of the top five countries in terms of planting rates (1) measured in hectares is also displayed, alongside potential outputs if all plantings had included EMF. The number of people supported is based on a daily calorie need of 2,250 per person and an annual calorific output per hectare of 316,206.72. All figures are millions.

## Results and Discussion

We estimate the potential of EMF cultivation by integrating production figures with current data on GHG flux of wooded systems (12). Combining data from 637,000 ground plots and 707,561 waveform light detection and ranging (LiDAR) observations alongside additional satellite data, each hectare managed as a tree crop in boreal systems may sequester around 12.8 t CO_2_e y^−^^1^, whereas in temperate ecosystems this is likely to be around 6.1 t CO_2_e y^−^^1^ and in tropical systems the flux may be very different with, instead, an emission rate of 3.8 t CO_2_e y^−^^1^ (Table 1). This difference is largely driven by the dataset incorporating carbon emissions in wooded environments such as commodity-driven deforestation, which is globally concentrated within the tropical climate domain (12). Although accurate for current socioeconomic environments, these figures may be a significant underrepresentation of carbon storage for productive EMF forests as these will likely be coveted by local beneficiaries and therefore less susceptible to deforestation. Using a different cultivation approach, akin to secondary forest creation, carbon sequestration across all habitat types is lessened and/or emissions increased for the first 20 y of establishment, after which the sequestration rate is increased in temperate and subtropical systems, with tropical systems becoming a net sink of 2.2 t CO_2_e y^−^^1^ rather than a source. If carbon sequestration is the goal, different approaches can therefore be used in different climate domains to maximize the potential. Going further, the GHG flux for different nutritional metrics can be calculated. For each kilogram of EMF protein produced, the GHG flux ranges from 526 to −858 kg CO_2_e y^−^^1^ (Table 2) and for each calorie the range is 24 to −39 g CO_2_e y^−^^1^ (Table 1). The positive figures are again largely driven by deforestation in the tropics (12), and so these flux estimates may be a significant underestimation of carbon sequestration potential. The comparison to other food items is stark (Table 2). For example, pulses are our most efficient production category but still present a net emission within the range of 4 to 10 GHG kg CO_2_-eq kg^−^^1^. Compared to our nine most important food categories, only EMF production can present a net sequestration of GHG during production. Viewed in terms of land use, EMF cultivation is less efficient, presenting a land use of 668 m^2^ y kg^−^^1^ protein. This is higher than all food production categories, aside from beef production which includes extensive pastoral systems with an upper figure of 2,100 m^2^ y kg^−^^1^ protein (Table 2). However, land-use figures should be interpreted in the context of EMF cultivation providing additional ecosystem services, GHG efficient food production and at the same time reducing the food-forestry land-use conflict. In this latter context, we have further explored the potential scale of such an approach. Current per-country afforestation rates, annualized for the period 2010 to 2020, have been published (1). Combining these with yield data and our nutritional calculations, we present the missed opportunity in terms of food production (Fig. 1).

**Table 1.** Average net greenhouse gas (GHG) flux for the most relevant forest types between 2001 and 2019 (after ref. 12) and subsequent estimates for net GHG flux per calorie and kg of protein produced from potential *L. deliciosus* cultivation

Climate domain	Forest type	Forest extent 2000 (Mha)	Net GHG flux GtCO_2_e y^−1^, 2001 to 2019	tCO_2_e y^−1^ ha^−^^1^	g CO_2_e y^−^^1^ per calorie	kg CO_2_e y^−1^ per kg protein
Boreal	Old secondary (>20 y)	1,030	−1.8	−1.7476	−5.2983	−116.6604
	Young secondary (≤20 y)	22	−0.022	−1	−3.0318	−66.7557
	Plantations/tree crops	0.21	−0.0027	−12.8571	−38.9804	−858.2872
Total boreal		1,052	−1.8247			
Temperate	Old secondary (>20 y)	560	−3.5	−6.25	−18.9488	−417.2230
	Young secondary (≤20 y)	16	0.0092	0.575	1.7433	38.3845
	Plantations/tree crops	12	−0.073	−6.0833	−18.4435	−406.0970
Total temperate		588	−3.5638			
Subtropical	Old secondary (>20 y)	270	−0.38	−1.4074	−4.2670	−93.9524
	Young secondary (≤20 y)	13	0.04	3.0769	9.3286	205.4021
	Plantations/tree crops	54	−0.31	−5.7407	−17.4048	−383.2270
Total subtropical		337	−0.65			
Tropical	Old secondary (>20 y)	880	−1.9	−2.1591	−6.5459	−144.1316
	Young secondary (≤20 y)	47	0.37	7.8723	23.8674	525.5234
	Plantations/tree crops	47	0.16	3.4043	10.3210	227.2534
Total tropical		974	−1.37			
Global	Old secondary (>20 y)	2,750	−7.7	−2.8	−8.4891	−186.9159
	Young secondary (≤20 y)	99	0.39	3.9394	11.9435	262.9769
	Plantations/tree crops	113	−0.23	−2.0354	−6.1709	−135.8744
Total global		2,962	−7.54			

**Table 2.** Average net greenhouse gas (GHG) flux and land use of key protein-rich food groups (after ref. 13) and estimates for *L. deliciosus* production

Product (%protein)	GHG kg CO_2_-eq kg^−1^ protein	Land use m^2^ y kg^−1^ protein
Beef (20%)	45 to 643	37 to 2,100
Pork (20%)	20 to 55	40 to 75
Poultry (20%)	10 to 30	23 to 40
Eggs (13%)	15 to 42	29 to 52
Mutton and lamb (20%)	51 to 750	100 to 165
Milk (3.5%)	28 to 43	26 to 54
Cheese (25%)	28 to 68	26 to 54
Seafood (16 to 20%)	4 to 540	–(on land 13 to 30)
Pulses, dry (20 to 36%)	4 to 10	10 to 43
*L. delicious*, dry (17.19%)	−858 to 526	668

Using the average suggested daily calorie intake per adult (2,250), we see that the forestry activities of China for the period 2010 to 2020 could have set in-place a production system capable of enough calorific output to support 4.6 million people annually (Fig. 1). Globally, with annualized planting rates of 4.7 million hectares, this presents a missed opportunity of enough calorific output to feed up to 18.9 million people, annually. Crucially, this may be achieved by only modifying the planting stock in current afforestation activities, and this can be a cost-effective process, using spore-based or cultured inoculum (4–6). Aside from a calorie and protein source, EMF are nutritionally valuable in terms of antioxidants and the provision of essential fatty acids, etc (14). Harvests may be consumed fresh, dried, or processed for protein extraction. However, not all tree species or planting scenarios will be suitable (6) and GHG flux will be influenced by stand management. These deficiencies may be balanced by the conservative yield estimates used here, and so the potential to increase afforestation activities at the same time as mitigating the farming-forestry land-use conflict remains high. Moreover, forests and trees in agroforestry systems provide an array of ecosystem services including water and microclimate regulation, shade and windbreak provision, soil protection, nutrient cycling, biological pest control, and pollination, alongside providing habitat for a diverse range of species that can reduce or reverse local biodiversity loss (1).

The technology is nascent, and more research is needed. Nevertheless, with such stark global potential we must urgently pursue such options, and we call for more researchers to join the field. We have highlighted the opportunity and the tools exist to take this forward; we need policy makers and research, funding, and development agencies to help make such options a reality.

## Materials and Methods

To estimate calorie and protein production, a yield figure of 1,089 kg ha^−^^1^ (5) for *L. deliciosus* cultivation (see *Results and Discussion* in main text) was combined with nutritional data. To calculate GHG flux of production, the most relevant forest types from published data were utilized (12). Full calculations, figures, and description of the data are detailed in *SI Appendix*.

## Supplementary Material

Appendix 01 (PDF)

## Data Availability

All study data are included in the article and/or *SI Appendix*.
