# Supplementary material for: Edible fungi crops through mycoforestry, potential for carbon negative food production and mitigation of food and forestry conflicts
Source: Proc Natl Acad Sci U S A. 2023 Mar 13;120(12):e2220079120. doi: 10.1073/pnas.2220079120 (PMC10041105; doi:10.1073/pnas.2220079120)
Supplement: Supplementary file 1 — Appendix 01 (PDF) [file pnas.2220079120.sapp.pdf]

PNAS

Supporting Information for

Edible fungi crops through mycoforestry, potential for carbon negative food production and mitigation of food and forestry conflicts

Paul W. Thomas[a,b,*] and Alistair S. Jump[a]

[a]Faculty of Natural Sciences, University of Stirling, FK9 4LA, Stirling, UK.

[b]Mycorrhizal Systems Ltd, Lancashire, PR25 2SD, UK

* Paul W. Thomas

Email: paul.thomas@stir.ac.uk

This PDF file includes:

Supporting text

Supporting Information Text

Extended methods

To estimate calorie production in EMF cultivation, a modest reported yield figure of 1,089 kg ha^{-1} [1] for *L. deliciosus* was used (see discussion in main text). *L. deliciosus* fruiting bodies contain 8% dry matter, with 379 kcal per 100 g dry weight[2]. Production of 1,089 kg/ha would equate to a calorie output of 329,800 kcal ha^{-1} y^{-1}. This figure is also used for GHG flux calculations. Based on an average suggested daily calorie intake per adult of 2,250 kcal (2,000 kcal for women, 2,500 kcal for men), 1 hectare of *L. deliciosus* production has the potential to provide enough calorific intake to sustain 0.4 average adults.

To calculate GHG flux of EMF production, the most relevant forest types from the published data was utilised[3]. This incorporated three categories: plantations and/or tree crops (for example, oil palm), secondary forests of below 20 years of age or thirdly, secondary forests above 20 years of age. Data from primary forests as well as mangroves, was deemed unsuitable for new EMF cultivation and was therefore excluded. The following equation was utilised:

$$\left(\left(\frac{\left(\frac{a}{b}\right) * 1000}{c}\right) * 1000 = d\right) * 1000 = e$$

Where *a* is the net GHG flux in $GtCO_2e\ yr^{-1}$, *b* is the extent of habitat type for the year 2000 (MHa) and *c* is the calories per Ha produced by *L. delicious* (329836.32). The resulting figure of $KgCO_2e\ yr^{-1}$ $calorie^{-1}$(*d*) is further used to calculate the $gCO_2e\ yr^{-1}$ $calorie^{-1}$ (*e*).

The calorific output of 329,836.32 kcal ha^{-1} yr^{-1} was used to calculate the amount of people such output could support.

L. deliciosus fruiting bodies contain 17.19g of crude protein per 100 g dry weight[2]. Using a yield of 1,089 kg ha^{-1} [1], this equates to 87.12kg dry weight production per hectare or 14.98kg of crude protein. At 1.498g/m^2, this represents a land use of 668 m^2 y kg^{-1} protein. This figure was further used to calculate $KgCO_2e\ yr^{-1}$ $KgProtein^{-1}$.

SI References

1. A. Guerin-Laguette, Successes and challenges in the sustainable cultivation of edible mycorrhizal fungi - furthering the dream. Mycoscience. 62(1), 10-28 (2021).
2. Z. Xu et al., Chemical Composition, Antioxidant and Antihyperglycemic Activities of the Wild Lactarius deliciosus from China. Molecules. 24(7), 1357 (2019). https://doi.org/10.3390/molecules24071357.
3. N. L. Harris *et al.,* Global maps of twenty-first century forest carbon fluxes. *Nat Clim Chang.* **11**(3), 234-240 (2021).
